# A Single‐Blind, Randomized, Clinical Trial Comparing Scalpel Versus Electrocautery for Skin Incision in Young Patients Undergoing Thyroidectomy

**DOI:** 10.1002/wjs.12651

**Published:** 2025-06-10

**Authors:** Jacqueline Hawthorne, Lokesh Sharma, Rosemary Carroll, Elizabeth Freihaut, Kirstin Miteff, Christine O'Neill, Cino Bendinelli

**Affiliations:** ^1^ Department of Surgery John Hunter Hospital New Lambton Heights Australia; ^2^ Department of Anaesthesia John Hunter Hospital New Lambton Heights Australia; ^3^ School of Medicine and Public Health University of Newcastle Callaghan Australia

**Keywords:** cosmesis, electrocautery, scalpel, thyroidectomy

## Abstract

Patient characteristics.

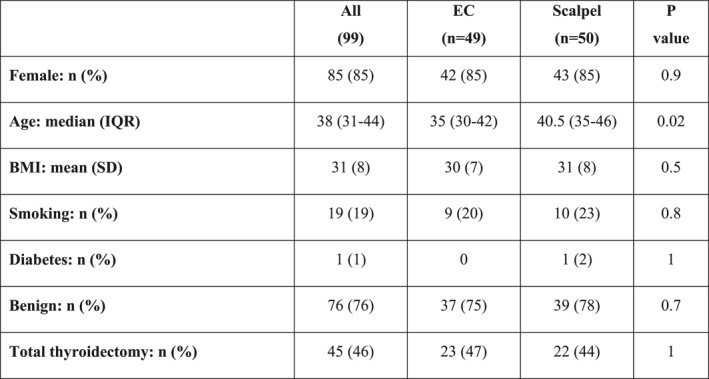

## 1 Introduction

Scalpel handling at the time of skin incision represents a well‐documented occupational hazard [1]. Monopolar electrocautery (EC) is routinely employed for the creation of subplatysmal flaps and further dissection and may well represent a more efficient and inherently safer alternative to scalpel. In randomized control trials (RCT) on laparotomy incisions, cosmetic outcomes were equivalent and postoperative pain reduced by EC when compared to scalpel [2, 3]. This study tested cosmesis and postoperative pain in young patients undergoing a cosmetically crucial neck incision for thyroidectomy.

## 2 Methods

A single‐blind RCT recruited patients aged 18–50 undergoing elective thyroid surgery. Those requiring neck dissection or with previous neck surgery or radiotherapy were excluded. Randomization dictated skin incision with either EC or scalpel. All procedures were carried out in standard fashion by two high volume endocrine surgeons (CB, CO). Perioperative analgesia was standardized.

The primary outcome was cosmesis at 6 months assessed with a modified patient and observer scar assessment scale 2.0 (POSAS) [4]. Participants scored pain, itch, color, stiffness, thickness, and irregularity using online surveys, with a total value ranging from 1 (optimal) to 60 (worst possible). A plastic surgeon scored scar photographs for vascularity, pigmentation, thickness, and surface area, with a total value ranging from 1 (optimal) to 40 (worst possible). Secondary outcomes were postoperative pain measured at 1 and 24 h with visual analog scale (VAS) and inhospital postoperative analgesic consumption measured in morphine milliequivalents. Wound related events were recorded. Both participants and plastic surgeon were blinded to treatment allocation.

Sample size was determined considering an expected mean POSAS of 11, smallest detectable difference of 2 mean points, power of 80%, and confidence interval of 95%. This was calculated to detect a 33% difference and account for a 10% drop out rate, giving a sample size of two groups of 40 patients each. A chi‐squared was used for categorical variables, independent *t*‐test was used for normally distributed data, and Mann–Whitney *U*‐test was used for skewed data. Statistical significance was set at *p* < 0.05.

## 3 Results

Between October 2018 and December 2023, 335 patients were referred for thyroid surgery and 99 were included in the study, with 49 randomized to EC and 50 to scalpel. Photographs and questionnaires were returned by 30 (61%) and 22 (44%) patients in EC and scalpel groups, respectively. The groups were statistically similar apart from a small age difference (Table 1).

**TABLE 1:** Patient characteristics.

	All (99)	EC (*n* = 49)	Scalpel (*n* = 50)	*p* value
Female: *n* (%)	85 (85)	42 (85)	43 (85)	0.9
Age: Median (IQR)	38 (31–44)	35 (30–42)	40.5 (35–46)	0.02
BMI: Mean (SD)	31 (8)	30 (7)	31 (8)	0.5
Smoking: *n* (%)	19 (19)	9 (20)	10 (23)	0.8
Diabetes: *n* (%)	1 (1)	0	1 (2)	1
Benign: *n* (%)	76 (76)	37 (75)	39 (78)	0.7
Total thyroidectomy: *n* (%)	45 (46)	23 (47)	22 (44)	1

Abbreviations: BMI, body mass index; EC, electrocautery; IQR, interquartile range; SD, standard deviation.

The cosmetic outcome did not differ between groups in either patient‐reported (18 vs. 17; *p* = 0.17) nor observer (10.5 vs. 9.5; *p* = 0.13) POSAS. Both postoperative VAS score and morphine milliequivalents used were similar (Table 2). One superficial wound dehiscence was observed in the EC group.

**TABLE 2:** POSAS, postoperative pain, and analgesic requirements. Lower scores are indicative of improved cosmesis.

	EC (*n* = 30)	Scalpel (*n* = 22)	*p* value
Patient POSAS (1–60)	18 (13–25)	17 (8.5–21.5)	0.17
Observer POSAS (1–40)	10.5 (9–14)	9.5 (8–13)	0.13
VAS at 1 h	4 (3–7)	5.5 (3–7)	0.41
VAS at 24 h	3 (1–4)	3 (1.5–5)	0.20
Morphine milliequivalent use	24 (16–40)	35 (19.5–55)	0.16

*Note:* Data reported as: median (interquartile range).

Abbreviations: EC, electrocautery; POSAS, patient and observers scar assessment score; VAS, visual analog scale.

## 4 Discussion

Thyroidectomy scars remain extremely noticeable and can effect patient quality of life [5]. A RCT on 19 bilateral neck dissections (38 incisions) performed with EC on one side and scalpel on the other showed no difference in cosmesis or patient satisfaction, but this study recruited patients with crucial confounders such as aggressive cancer, advanced age, and/or radiotherapy [6]. Geometric electron modulation (GEM) EC offers similar hemostasis to EC with reduced heat exchanged to surrounding tissues [7]. GEM EC was compared to scalpel in a small RCT on 11 thyroidectomy and 11 lateral neck dissections. No statistically significant differences were observed in cosmetic outcomes at 3 months, but this study evaluated a slightly different technology and premature scars [8].

In this trial, we focused on a younger population and excluded patients with locally advanced disease or prior neck surgery. The cosmetic outcome was assessed blindly at 6 months by both the patients and a plastic surgeon. Cosmetic outcomes were generally good, and patients of both groups were equally satisfied with cosmesis. Observer scores were also comparable. The fascinating assumption, that nerve necrosis by EC tissue vaporization may reduce postoperative pain, could not be validated, potentially due to the small series.

The main study limitations were related to the COVID‐19 pandemic and included a longer than expected study period and a lower than expected patient response rate. This might be responsible for a type II error. Furthermore, the length of the incision, which may influence both cosmesis and pain, was not collected. Thyroidectomy incisions do vary depending on the gland size, but the randomization process would have eliminated this potential bias.

In conclusion, we demonstrated that using EC rather than scalpel to perform the skin incision leads to comparable cosmetically outcomes even in the neck of young patients. We encourage this practice to optimize occupational safety.

## Author Contributions

**Jacqueline Hawthorne:** data curation, formal analysis, writing – original draft. **Lokesh Sharma:** investigation, writing – original draft. **Rosemary Carroll:** writing – original draft, data curation. **Elizabeth Freihaut:** methodology, writing – original draft. **Kirstin Miteff:** investigation. **Christine O’Neill:** writing – review and editing. **Cino Bendinelli:** conceptualization, supervision, writing – review and editing.

## Ethics Statement

Hunter New England Local Health District Human Research Ethics Committee: 2018/ETH00013.

## Conflicts of Interest

The authors declare no conflicts of interest.

## Data Availability

The data that support the findings of this study are available from the corresponding author upon reasonable request.
